# Evaluation of sleep quality and pruritus severity in psoriatic patients and their impact on quality of life: a cross section correlational study

**DOI:** 10.1038/s41598-023-44757-5

**Published:** 2023-10-16

**Authors:** Mohamed S. Zaky, Emad Eldin A. Elgamal, Ayman A. Abd Al Maksoud, Dina H. Mohamed, Mohamed L. Elsaie

**Affiliations:** 1 Department of Dermatology, Venereology and Andrology, Damietta Faculty of Medicine, Al-Azhar University, Damietta, Egypt; 2 Department of Psychiatry, Damietta Faculty of Medicine, Al-Azhar University, Damietta, Egypt; 3 Department of Dermatology, Venereology and Andrology, Medical Research and Clinical Studies Institute, National Research Centre, Giza, Egypt

**Keywords:** Medical research, Epidemiology

## Abstract

Psoriasis is a chronic inflammatory condition associated with genetic and immunological susceptibility. The objective of the study was to evaluate pruritus and sleep quality in correlation (r) to psoriasis severity and to detect their impact on quality of life. Two hundred (200) patients with psoriasis were included. Psoriasis severity was determined using the psoriasis area severity index (PASI), the quality of life (QoL) was assessed by the psoriasis disability index (PDI) questionnaire, and the sleep quality was evaluated by the Pittsburgh sleep quality index (PSQI). Finally, the severity of itching was evaluated using a 12-item pruritus severity scale (PSS). Poor sleep quality was found in 16.0% of patients in this study. Poor sleep was detected among 50.0% of cases with severe psoriasis. PASI scores correlated significantly with sleep quality, duration and sleep disturbances (p < 0.001). The global PSQI and PASI were also significantly correlated (p = 0.004). In conclusion patients complaining of psoriasis exacerbated by pruritus and sleep problems demonstrated lower quality of life in all domains. Sleep disturbances and depressive symptoms impairing quality of life should be taken into consideration when screening patients suffering from psoriasis.

## Introduction

Psoriasis is a chronic inflammatory skin disease with genetic and immunological background and is exacerbated and triggered by environmental factors^1^. Sleep disturbances (SDs) are experienced by psoriatic patients and affect their quality of life. A close relationship exists between psoriasis and SDs, the pathophysiological mechanisms of which are not fully understood.

Psoriasis is characterized by activation of T-helper cells (Th-1), antigen presenting cells (APCs) and by recruitment of Th-1 cytokines characterized by expansion and activation of T-helper cells (Th-1), antigen presenting cells, and Th-1 cytokines and interleukins. It had been revealed that increased serum levels of inflammatory cytokines such as tumor necrosis factor alpha and interleukin 6 are responsible for sleep restriction in psoriasis^2,3^. Moreover; higher concentration of substance P discovered in perilesional skin of psoriatic patients had been related to disturbed sleep cycles and their regulation^4^. In addition to the above, the lower melatonin levels expressed in serum of psoriatic patients can directly influence sleep habits and potentially be responsible for increased fatigue^5^.

Pruritus is known to negatively impact sleep in patients with other dermatological conditions including chronic urticaria, chronic pruritus (CP) and atopic dermatitis (AD). Pruritus severity in patients with psoriasis is lower than that in AD or CP but it often intensifies in the evening, thereby potentially interfering with sleep^6^.

Pruritus defined as an unpleasant sensation that provokes a desire to scratch. The molecular basis of pruritus in psoriasis is still not fully elucidated, albeit a complex interaction between the nervous, neuroendocrine, immune, and vascular systems is suggested. Many mediators were indicated to modulate this sensation in psoriasis, but none has been proven to be a crucial one to date^6^.

Contradictory findings exist regarding the association of pruritus intensity with sleep disturbance among psoriatic patients. The coexisting relation of SD’s among psoriatic patients is multifactorial and its exact pathophysiologic mechanism remains to be fully elucidated. The purpose of this study was to evaluate pruritus and sleep quality in correlation (r) to psoriasis severity and to detect their impact on quality of life.

## Patients and methods

This study was a cross sectional study that included 200 patients diagnosed clinically with psoriasis that were recruited from the outpatient clinics. Twenty three subjects were excluded earlier due to being on topical or systemic medications at the time of data collection. Following the study’s approval by the local ethics committee at the Faculty of Medicine at Al-Azhar University in Damietta (IRB 00012367-21-03-003), all cases included in the study provided their verbal or written informed consent.

Patients were included if they had established psoriasis of more than 6 months and were able to read and write for completing the questionnaire. Exclusion criteria included pregnant or lactating females, other concomitant dermatological or systemic disorders that might cause pruritus such as chronic uremia or cholestasis, and usage of medications that could influence the sensation of itch. Moreover Subjects with cognitive impairment, present psychiatric disorders, active depression, substance use, and smoking as well as those pustular psoriasis and cases with concomitant psoriatic arthritis (PsA) were also excluded.

Every patient was subjected to:

(I) Questioning about:
  1. Age.
  2. Gender.
  3. Smoking and substance use.
  4. Any coexistent chronic diseases (diabetes mellitus, hypertension, heart disease, chronic kidney disease, chronic liver disease, thyroid disease, stroke).
  5. Drugs such as (Opioids, Hypnotics and sedatives, thyroid hormones).
  6. Disease duration.
(II) Dermatological examination:
  - To exclude dermatological diseases other than psoriasis.
  - Assessment of psoriasis according to clinical types.
(III) *Psoriasis grading with Psoriasis Area Severity Index (PASI)* PASI score was used for severity and extent assessment of psoriasis. It ranges from zero (no disease) to 72 (severe disease) (maximal disease severity. Scores below 10 mark mild psoriasis and scores above 20 are regarded as severe while scores falling between 10 and 20 are regarded as moderate psoriasis (6).
(IV) *Evaluation of Disease-related Quality of Life of the subjects using Arabic version of Psoriasis Disability Index (PDI)* The effect of psoriasis on daily activities, work or school, relationships, leisure, and treatment were assessed using the psoriasis disability index questionnaire (PDI)^7^. The reliability of the Arabic version of the DPI was validated and was found to be reliable for evaluating the QOL for Egyptian patients with psoriasis and was used in the current study^8^.
  The PDI is made up of 15 simple, disease-related questions. Each question is scored on a scale of 0 to 4, with a maximum score of 45. The lower the score, the lower is the quality of life. The PDI can also be reported as a percentage of a maximum possible score of 45. The overall score indicates how psoriasis has affected the patient over the last four weeks.
(V) *Evaluation of sleep quality using arabic translation of Pittsburgh sleep quality index (PSQI)* Sleep quality was assessed using the Arabic validated version^9^ of the PSQI^10^. Seven components are used; subjective sleep quality, latency of sleep, duration of sleeping, habitual sleep efficiency, disturbances of sleep, whether or not sleep medications are used, and dysfunction of the following day over the last month. Each component is scored from 0 to 3, resulting in a global PSQI score between 0 and 21, with higher scores indicating lower quality of sleep. The PSQI score ≥ 5 was considered as a cut-off for poor sleep quality.
(VI) *Assessment of pruritus severity using 12-item pruritus severity scale (PSS)* The 12-PSS is a one page assessment tool assessing different aspects of pruritus. Five domains namely: pruritus intensity, pruritus extent, frequency and duration of pruritus, impact of pruritus on daily activities and mood, and assessment of scratching. Participants answered 12 questions referring to their pruritus and the total scoring ranged from 3 (the lowest pruritus intensity) to 22 points (the highest pruritus intensity). According to scores pruritus is classified as mild pruritus (3–6 points) moderate pruritus (7–11 points) or severe pruritus (12–22 points)^11^.

### Sample size calculation

Sample size calculation was based on 90% bad sleep quality among cases with psoriasis retrieved from a previous study by Saçmacı and Gurel^12^. With a 95.0% Confidence interval and an acceptable margin error of 5.0%, the calculated sample size in the study was determined to be at least 138 participants. Taking into consideration expected dropouts of 20.0%, an approximate sum of 166 participants was determined to be sufficient.

### Statistical analysis

The data was anonymized and fed to personal computer software statistical package of social sciences (SPSS), version 16 (SPSS Inc., Chicago, USA). Data had been presented by their mean, median, standard deviation, relative frequency and percentages according to its type and normality of distribution. Groups compared by independent samples “t” test, Chi square test or any equivalent according to type of data. Pearson’s correlation (r) coefficient was calculated to address possible correlation (r) between variables and p value < 0.05 was set as statistically significant.

### Study approval statement

This study protocol was reviewed and approved by ethics committee on human research by Al Azhar Damietta faculty of medicine (No. 00012367-21-03-003). All methods were performed in accordance with the relevant guidelines and regulations.

### Consent to participate statement

Written informed consents were received from participants upon explanation of the study. Consent for publication were obtained from the participants for publishing the images in the manuscript.

## Results

The mean age of the studied cases was 38.9 ± 17.84 years ranging from 6 to 75 years among which half of them were females. One hundred and fifty six cases (78.0%) were diagnosed as plaque type psoriasis, 32 cases (16.0%) suffered from palmo plantar psoriasis and only 12 (6.0%) of cases had scalp psoriasis. The median disease duration was 7.3 ± 6.2 years and ranged from 1 to 30 years.

The median PASI score was 5.6 and ranged from 0.5 to 22.8. Of the studied cases; 76% had mild PASI, 20.0% demonstrated moderate PASI and only 4.0% presented with severe PASI. The pruritus severity scale ranged from 3 to 20 with a median score of 9; with 34.0% of cases presenting with mild pruritus, 32.0% with moderate pruritus and 34.0% with severe pruritus. Psoriasis disability index ranged from 0 to 26 with a median score of 5. Poor sleep quality was found in 16.0% of patients in this study (Tables 1, 2).

**Table 1:** PASI score and pruritus severity scale and psoriasis disability index distribution among studied cases.

	n = 200	%
PASI score	5.6 (0.5–22.8)	
Mild < 10	152	76.0
Moderate (10–20)	40	20.0
Severe > 20	8	4.0
Pruritus severity scale	9 (3–20)	
Mild (3–6)	68	34.0
Moderate (7–11)	64	32.0
Severe (12–22)	68	34.0
Psoriasis disability index		
Median (min–max)	5 (0–26)	

*PASI* psoriasis area severity index, *n* number, *%* percentage.

**Table 2:** Distribution of sleep quality scores among studied cases.

PSQI components	n	%
Subjective sleep quality		
0	138	69.0
1	50	25.0
2	12	6.0
Sleep latency		
0	104	52.0
1	62	31.0
2	16	8.0
3	18	9.0
Sleep duration		
0	150	75.0
1	44	22.0
2	4	2.0
3	2	1.0
Sleep efficiency		
0	150	75.0
1	42	21.0
2	6	3.0
3	2	1.0
Sleep disturbance		
0	116	58.0
1	76	38.0
2	8	4.0
Use of sleeping medications		
0		
1	0	0.0
2		
Day time dysfunction		
0	172	86.0
1	24	12.0
2	4	2.0
Global score	2 (0–8)	
Good sleep ≤ 5	168	84.0
Poor sleep > 5	32	16.0

*PSQI* Pittsburgh sleep quality index, *n* number, *%* percentage.

Among all sleep quality domains, a statistically significant relation was established between sleep efficiency and the type of psoriasis (p = 0.036). A sleep efficiency score of 0 was detected among 56.2% of cases with palmoplantar psoriasis versus 78.2% of cases with plaque type psoriasis and 83.3% of cases with scalp psoriasis. Scores of 2 and 3 among the efficiency domain was only detectable among cases with plaque type psoriasis when compared to subjects with palmpolantar psoriasis or scalp psoriasis. Sleeping efficiency was significantly lower among subjects with plaque type psoriasis when compared to the other two groups (p = 0.036). The median disease duration among cases with poor sleep was higher than cases with good sleep (10 years vs. 6.5 years; p = 0.039). Longer disease duration was associated with significantly lower quality of sleep in all domains (Tables 3, 4).

**Table 3:** Relation between psoriasis type and sleep quality in studied cases.

PSQI components	Psoriasis type			Test of significance
	Palmoplantar psoriasis n (%)	Plaque type psoriasis n (%)	Scalp psoriasis n (%)	
Subjective sleep quality				
0	22 (68.8)	104 (66.7)	12 (100)	MC = 5.79 p = 0.215
1	8 (25)	42 (26.9)	0	
2	2 (6.2)	10 (6.4)	0	
Sleep latency				
0	14 (43.8)	80 (51.3)	10 (83.3)	MC = 6.86 p = 0.334
1	10 (31.2)	50 (32.1)	2 (16.70	
2	4 (12.5)	12 (7.7)	0	
3	4 (12.5)	14 (9.00	0	
Sleep duration				
0	24 (75)	116 (74.4)	10 (83.3)	MC = 4.61 p = 0.595
1	6 (18.8)	36 (23.1)	2 (16.7)	
2	2 (6.2)	2 (1.3)	0	
3	0	2 (1.3)	0	
Sleep efficiency				
0	18 (56.2)	122 (78.2)	10 (83.3)	MC = 13.47 p = 0.036*
1	14 (43.8)	26 (16.7)	2 (16.7)	
2	0	6 (3.8)	0	
3	0	2 (1.3)	0	
Sleep disturbance				
0	18 (56.2)	90 (57.7)	8 (66.7)	MC = 2.81 p = 0.591
1	14 (43.8)	58 (37.2)	4 (33.3)	
2	0	8 (5.1)	0	
Day time dysfunction				
0	30 (93.8)	132 (84.6)	10 (83.3)	MC = 2.58 p = 0.630
1	2 (6.2)	20 (12.8)	2 (16.7)	
2	0	4 (2.6)	0	
Global score				
Good sleep ≤ 5	26 (81.7)	130 (83.3)	12 (100)	MC = 2.52 p = 0.284
Poor sleep > 5	6 (18.8)	26 (16.7)	0	

*MC* Monte Carlo test, *PSQI* Pittsburgh sleep quality index, *n* number, *%* percentage.

*Statistically significant.

**Table 4:** Relation between psoriasis duration and sleep quality in studied cases.

PSQI components	Disease duration/years Median (min–max)	Test of significance
Subjective sleep quality		
0	6 (1–30)	KW = 12.6 p = 0.002*
1	10 (3–30)	
2	9.5 (2–12)	
Sleep latency		
0	6 (1–30)	KW = 16.79 p = 0.001*
1	7 (1–20)	
2	9 (3–30)	
3	11 (1–20)	
Sleep duration		
0	7 (1–20)	KW = 10.0 p = 0.019*
1	9 (1–30)	
2	11.5 (7–16)	
3	17 (17–17)	
Sleep efficiency		
0	7 (1–30)	KW = 8.61 p = 0.035*
1	9 (1–20)	
2	12 (2–18)	
3	3 (3–3)	
Sleep disturbance		
0	7 (1–20)	KW = 6.88 p = 0.032*
1	8 (1–30)	
2	9 (4–10)	
Day time dysfunction		
0	7 (1–30)	KW = 6.49 p = 0.039*
1	10 (1–17)	
2	9 (9–9)	
Global score		
Good sleep ≤ 5	6.5 (1–30)	Z = 3.41 p = 0.001*
Poor sleep > 5	10 (2–18)	

*KW* Kruskal Wallis test, *Z* Mann Whitney U test, *PSQI* Pittsburgh sleep quality index.

*Statistically significant.

Poor sleep was detected among 50.0% of cases with severe psoriasis, 25.0% with moderate psoriasis and 11.8% subjects complaining of mild psoriasis. The scores of subjective sleep quality, sleep duration, habitual sleep efficiency, sleep disturbance and daytime dysfunction had significant correlation (r)s with PASI (p < 0.001). The global PSQI and PASI were also significantly correlated (p = 0.004). Cases with severe pruritus were associated with higher incidence of sleep disturbance in all domains (p < 0.001). The global PSQI and pruritus severity were also significantly correlated (p < 0.001). A median higher psoriasis disability index percent was associated with poor sleep than cases with good sleep (20.5% vs. 4.0%). The lower global PSQI was significantly associated with more psoriasis disability (p < 0.001) (Tables 5, 6, 7, 8).

**Table 5:** Relation between PASI score and sleep quality in studied cases.

PSQI components	PASI score			Test of significance
	Mild < 10 N (%)	Moderate (10–20) N (%)	Severe > 20 N (%)	
Subjective sleep quality				
0	112 (73.7)	22 (55.0)	4 (50.0)	MC = 20.56 p < 0.001*
1	36 (23.7)	10 (25.0)	4 (50.0)	
2	4 (2.6)	8 (20.0)	0	
Sleep latency				
0	84 (55.3)	16 (40)	4 (50)	MC = 8.63 p = 0.196
1	40 (26.3)	18 (45)	4 (50)	
2	12 (7.90	4 (10)	0	
3	16 (10.5)	2 (5)	0	
Sleep duration				
0	112 (73.7)	32 (80)	6 (75)	MC = 51.46 p < 0.001*
1	36 (23.7)	8 (20)	0	
2	4 (2.6)	0	0	
3	0	0	2 (25)	
Sleep efficiency				
0	114 (75)	32 (80)	4 (50)	MC = 59.89 p < 0.001*
1	36 (23.7)	4 (10)	2 (25)	
2	2 (1.3)	4 (10)	0	
3	0	0	2 (25)	
Sleep disturbance				
0	82 (53.9)	26 (65.0)	8 (100)	MC = 46.40 p < 0.001*
1	70 (46.1)	6 (15.0)	0	
2	0	8 (20.0)	0	
Day time dysfunction				
0	136 (89.5)	32 (80)	4 (50)	MC = 27.62 p < 0.001*
1	16 (10.5)	4 (10)	4 (50)	
2	0	4 (10)	0	
Global score				
Good sleep ≤ 5	134 (88.2)	30 (75)	4 (50)	MC = 11.25 p = 0.004*
Poor sleep > 5	18 (11.8)	10 (25)	4 (50)	

*MC* Monte Carlo test, *PSQI* Pittsburgh sleep quality index, *PASI* psoriasis area severity index, *n* number, *%* percentage.

*Statistically significant.

**Table 6:** Relation between PSS score and sleep quality in studied cases.

PSQI components	PSS score			Test of significance
	Mild (3–6) n (%)	Moderate (7–11) n (%)	Severe (12–22) n (%)	
Subjective sleep quality				
0	66 (97.1)	48 (75)	24 (35.3)	MC = 69.05 p < 0.001*
1	2 (2.9)	16 (25)	32 (47.1)	
2	0	0	12 (17.6)	
Sleep latency				
0	58 (85.3)	36 (56.2)	10 (14.7)	MC = 87.21 p < 0.001*
1	10 (14.7)	24 (37.5)	28 (41.2)	
2	0	2 (3.1)	14 (20.6)	
3	0	2 (3.1)	16 (23.5)	
Sleep duration				
0	62 (91.2)	52 (81.2)	36 (52.9)	MC = 32.58 p < 0.001*
1	6 (8.8)	12 (18.8)	26 (38.2)	
2	0	0	4 (5.9)	
3	0	0	2 (2.9)	
Sleep efficiency				
0	56 (82.4)	52 (81.2)	42 (61.8)	MC = 19.43 p = 0.003*
1	12 (17.6)	12 (18.8)	18 (26.5)	
2	0	0	6 (8.8)	
3	0	0	2 (2.9)	
Sleep disturbance				
0	52 (76.5)	42 (65.6)	22 (32.4)	MC = 19.43 p = 0.003*
1	14 (20.6)	20 (31.2)	42 (61.8)	
2	2 (2.9)	2 (3.1)	4 (5.9)	
Day time dysfunction				
0	66 (97.1)	58 (90.6)	48 (70.6)	MC = 23.35 p < 0.001*
1	2 (2.9)	4 (6.2)	18 (26.5)	
2	0	2 (3.1)	2 (2.9)	
Global score				
Good sleep ≤ 5	68 (100)	64 (100)	36 (52.9)	MC = 73.95 p < 0.001*
Poor sleep > 5	0	0	32 (47.1)	

*MC* Monte Carlo test, *PSQI* Pittsburgh sleep quality index, *PSS* Pruritus severity score, *n* number, *%* percentage.

*Statistically significant.

**Table 7:** Relation between psoriasis disability index and sleep quality in studied cases.

PSQI components	Psoriasis disability index	Test of significance
Subjective sleep quality		
0	3 (0–26)	KW = 61.88 p < 0.001*
1	8 (1–26)	
2	19 (4–21)	
Sleep latency		
0	3 (0–26)	KW = 53.90 p < 0.001*
1	8 (0–26)	
2	12 (1–26)	
3	10 (0–21)	
Sleep duration		
0	4 (0–26)	KW = 22.51 p < 0.001*
1	7.5 (0–26)	
2	21.5 (20–23)	
3	23 (23–23)	
Sleep efficiency		
0	4 (0–26)	KW = 26.02 p < 0.001*
1	10 (0–26)	
2	7 (4–15)	
3	26 (26–26)	
Sleep disturbance		
0	4 (0–26)	KW = 23.29 p < 0.001*
1	19.5 (0–26)	
2	20 (17–21)	
Day time dysfunction		
0	4 (0–26)	KW = 38.74 p < 0.001*
1	19 (0–26)	
2	16.5 (12–21)	
Global score		
Good sleep ≤ 5	4 (0–26)	Z = 7.44, p < 0.001*
Poor sleep > 5	20.5 (4–26)	

*KW* Kruskal Wallis test, *Z* Mann Whitney U test, *PSQI* Pittsburgh sleep quality index.

*Statistically significant.

**Table 8:** Factors affecting sleep quality in studied cases.

	Good sleep ≤ 5 n (%)	Poor sleep > 5 n (%)	Test of significance
Age/years			
Mean ± SD	38.74 ± 18.76	39.75 ± 11.67	t = 0.294 p = 0.769
Sex			
Male	82 (48.8)	18 (56.2)	χ^2^ = 0.595 p = 0.440
Female	86 (51.2)	14 (43.8)	
Psoriasis type			
Palmoplantar psoriasis	26 (15.5)	6 (18.8)	χ^2^ = 2.52 p = 0.284
Plaque type psoriasis	130 (77.4)	26 (81.2)	
Scalp psoriasis	12 (7.1)	0	
Disease duration/years			Z = 3.41 p = 0.001*
Median (min–max)	6.5 (1–30)	10 (2–18)	
PASI score			
Mild < 10	134 (79.8)	18 (56.2)	χ^2MC^ = 11.25 p = 0.004*
Moderate (10–20)	30 (17.9)	10 (31.2)	
Severe > 20	4 (2.4)	4 (12.5)	
Pruritus severity scale			
Mild (3–6)	68 (40.5)	0	MC = 73.95 p < 0.001*
Moderate (7–11)	64 (38.1)	0	
Severe (12–22)	36 (21.4)	32 (100)	
Psoriasis disability index%	4 (0–26)	20.5 (4–26)	Z = 7.44 p < 0.001*

*MC* Monte Carlo test, *MC* Monte Carlo test, *χ*^*2*^ Chi-square test, *Z* Mann Whitney U test, *PASI* psoriasis area severity index, *n* number, *%* percentage.

*Statistically significant.

**Figure 1:**
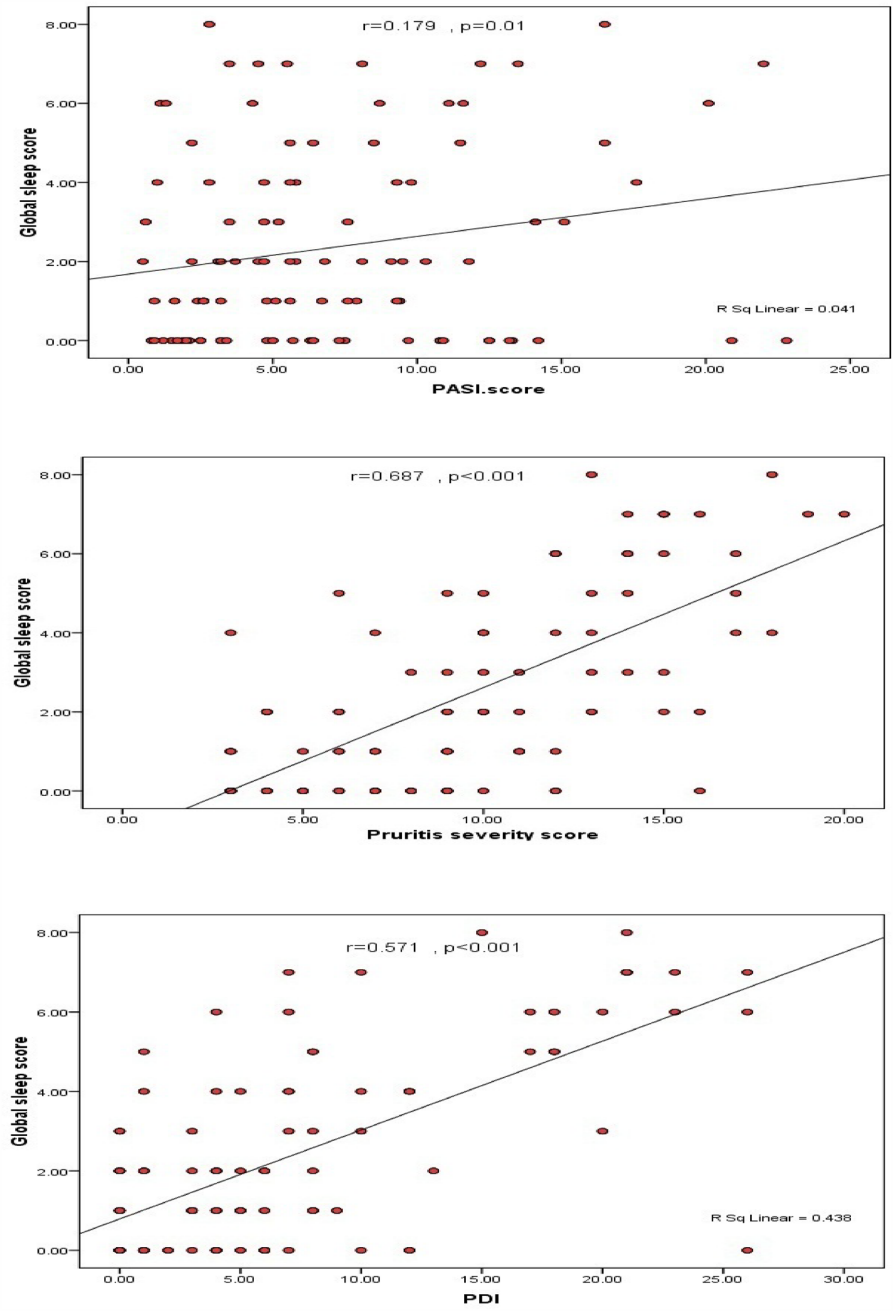
Scatter diagram showing positive correlation between PASI, PSS, PDI and sleep quality.

PSS and PDI were significant predictors of poor sleep quality (r = 0.687; r = 0.571; p < 0.001 respectively). According to linear regression analysis; disease duration, PSS and PDI were significant predictors of global sleep score with 64.8% of the score predicted by the 3 variables and the calculated prediction equation (Global score =  − 10.09 + 0.067 × disease duration + 0.242 × PSS + 0.150 × PDI) (Tables 9, 10, Fig. 1).

**Table 9:** Predictors of poor sleep in studied cases.

	β	p value	Odds ratio (95% CI)
Disease duration/years	0.061	0.217	1.06 (0.965–1.17)
PASI score	− 0.045	0.481	0.956 (0.842–1.08)
PSS	0.454	< 0.001*	1.57 (1.26–1.97)
PDI	0.238	< 0.001*	1.27 (1.14–1.41)
Overall % predicted = 92.0%; Model χ^2^ = 108.88; p < 0.001*			

*PASI* psoriasis area severity index, *PSS* Pruritus severity score, *PDI* Psoriasi disability index, *CI* confidence interval, *β* regression coefficient.

*Statistically significant.

**Table 10:** Linear regression for prediction of global sleep score.

	β	t	p value
Disease duration/years	0.067	3.788	< 0.001*
PASI score	− 0.037	− 1.604	0.110
PSS	0.242	9.113	< 0.001*
PDI	0.150	7.988	< 0.001*
Prediction equation	Global score = − 10.09 + 0.067 × disease duration + 0.242 × PSS + 0.150 × PDI		
F = 89.59, p < 0.001*; Constant = − 1.24; R^2^ = 0.648			

*PASI* psoriasis area severity index, *PSS* pruritus severity score, *PDI* psoriasi disability index, *β* regression coefficient, *t* t test.

*Statistically significant.

## Discussion

The current study showed that 16.0% of psoriatic subjects included reported poor sleep quality. Moreover; we determined a significant impact of psoriasis severity and pruritus on the disability index and quality of sleep.

Different studies investigated the reciprocal effect of sleep impairment and its relation to psoriasis or its severity using different assessment tools such as the PSQI, the Functional Outcomes of Sleep Questionnaire (FOSQ), the General Sleep Disturbance Scale (GSDC) and the general health questionnaire (GHQ-H)^12–35^ (Table 11).

**Table 11:** Prevalence of sleep disorders (SDs) among psoriatic patients in different studies.

Study	Year	Total number of patients	Prevalence of SDs (%)	Method for evaluating SDs
Sharma et al.^13^	2001	30	56.7	GHQ-H
Delfino et al.^14^	2008	40	55	Interview
Stiens et al.^15^	2008	135	34	Interview
Callis Duffin et al.^16^	2009	420	49.5	Interview
Hu et al.^17^	2010	100	60	Interview
Tsai et al.^18^	2011	51,800	0.05	Interview
Ljosaa et al.^19^	2012	139	62.6	GSDS
Shutty et al.^20^	2013	35	77.1	PSQI
Nyunt et al.^21^	2013	223	40.9	Interview
Sanchez-Carazo et al.^22^	2014	1022	12.2	Interview
Chiu et al.^23^	2016	99,628	2.2	Interview
Melikoglu ^24^	2017	58	60.3	PSQI
Henry et al.^25^	2017	186	76.8	PSQI
Wong et al.^26^	2017	62	69.3	PSQI
Jensen et al.^27^	2018	179	53.6	PSQI
Krajewska-Włodarczyk et al.^28^	2018	52	57.7	PSQI
Saçmacı and Gurel ^12^	2019	60	90	PSQI
Duvetorp et al.^29^	2019	911	16.3	Interview
Hawro et al.^30^	2020	104	39.4	DLQI
Luca et al.^31^	2020	83	83.9	PSQI
Nowowiejska et al.^32^	2021	60	78.3	PSQI
Jaworecka et al.^33^	2022	295	39.3	DLQI
Sahin et al.^34^	2022	334	59	PSQI
Khalaf et al.^35^	2023	100	38	PSQI

*PSQI* Pittsburgh sleep quality index, *GSDS* general sleep disturbance scale, *GHQ-H* general health questionnaire, *DLQI* dermatology life quality index (DLQI).

Sleep disturbances (SDs) were found to be higher among patients with psoriatic arthritis (45.1%) when compared to patients with psoriasis (16.0%)^29^. The current study confirmed previous reports demonstrating that psoriasis severity is associated with increased risk of having sleep disturbances (SDs)^20,24,36^. Melikoglu found out that in 48 recruited patients of psoriasis PSQI and SDs significantly correlated with PASI scores (p = 0.03)^24^.

Samaci et al. and Khalaf et al. recruited 60 and 100 patients complaining of psoriasis with a mean PASI score of (10.1 ± 9.7) and (4.97 ± 5.24) respectively. On the contrary to our results, both authors did not find any correlation (r) between either PASI severity or psoriasis duration and the degree of sleep affection^12,35^. The small percentage of poor sleep quality in the current research could be explained by the fact that mild disease was reported in the majority of our patients. Nevertheless; we detected a significant (p-value = 0.008) positive correlation (r) (r = 0.37) between sleep disturbances and PASI severity. Jaworecka et al. demonstrated that 39.3% (*n* = 116) of patients reported occasional difficulties in falling asleep, and 22.7% of them (*n* = 67) had such problems almost every day. Moreover, 20.3% (*n* = 60) woke up during sleep almost every night, and a further 33.6% (*n* = 99) reported such problem sporadically^33^. A study by Nowowiejska et al. reported poor sleep quality, was noticed in 47 patients (78.3%), 24 patients (40.0%) subjectively assessed their sleep quality as fairly bad or bad^32^.

Pruritus is characterized by uncomfortable burning sensation, numbness or even pain. Psoriatic patients with prutiris experienced less sleep quality and more sleep disturbances and daytime dysfunction than those patients without pruritus. Pruritus intensity correlated with PASI severity and cases with severe pruritus were associated with higher incidence of sleep disturbance in all domains (p < 0.001).

Studies determined that psoriatic patients complaining of pruritus show difficulty in falling asleep and have an increased frequency of sleep fragmentation and nocturnal awakening^16,37^. Meanwhile it was established that good sleep significantly reduces the intensity of itching related to psoriasis^33,38,39^.

In the current study patients with higher psoriasis disability index (PDI) had significantly diminished components of all sleep components and daytime dysfunction than those patients with lower score of PDI (p < 0.001). We also observed that total PDI was significantly higher among those subjects with moderate to severe psoriasis when compared to those with mild psoriasis (9.5 vs. 4.0, p < 0.001). Severity of psoriasis had been linked to impaired Quality of life (QoL) in a number of studies^22,40^.

Among the limitations of the current study are the cross-sectional design and the absence of a healthy control group. Uneven distribution of patients with varying severity of skin lesions represents another limitation as the majority of cases were of mild PASI score. In addition, sleep quality was assessed by means of a questionnaire while a polysomnographic evaluation could have yielded more accurate results.

In conclusion, we demonstrated that psoriatic patients with pruritus and sleep problems had a worse overall quality of life in all domains. Sleep impairment and screening of any depressive and impaired quality of life symptoms shall be considered when treating psoriatic patients.

## Data Availability

The data that support the findings of this study are available from the corresponding author upon reasonable request.

## Abbreviations

PASI: Psoriasis area severity index
QoL: Quality of life
PDI: Psoriasis disability index
PSQI: Pittsburgh sleep quality index
PSS: Pruritus severity scale
Th-1): T-helper cells
APCs: Antigen presenting cells
